# Important Aspects of siRNA Design for Optimal Efficacy In Vitro and In Vivo

**DOI:** 10.1155/ijcb/6663816

**Published:** 2025-12-17

**Authors:** Mili S. Bhakta-Yadav, Thomas L. Brown

**Affiliations:** ^1^ Department of Pharmacology and Toxicology, Wright State University Boonshoft School of Medicine, Dayton, Ohio, USA, wright.edu; ^2^ Department of Neuroscience, Cell Biology and Physiology, Wright State University Boonshoft School of Medicine, Dayton, Ohio, USA, wright.edu

**Keywords:** gene knockdown, messenger RNA (mRNA), nucleotide chemical modifications, RNA interference (RNAi), short-interfering RNA (siRNA), siRNA design

## Abstract

siRNA is a versatile tool with practical applications in various fields, such as fundamental research, therapeutic development, and plant genetics. A few siRNA therapeutics have been FDA‐approved, such as Onpattro (patisiran) and Leqvio (inclisiran) to treat hereditary transthyretin amyloidosis and primary hypercholesterolemia, respectively. In addition, several others are currently in clinical trials, highlighting the potential of siRNA‐based treatment for undruggable targets. siRNA is a double‐stranded RNA molecule that has the potential to inhibit gene expression by degrading target mRNA. The siRNA sequence must be precisely designed for effective gene knockdown and to minimize off‐target effects. Strategies for designing siRNA to achieve optimal efficacy are presented in this review. We emphasize approaches that promote effective gene knockdown by siRNA. These approaches include preventing off‐target RNAi and ensuring incorporation of the intended guide/antisense strand into RISC for targeted gene knockdown. This review also discusses the assessment of siRNA efficacy in vitro and the design of appropriate nonsilencing controls. Furthermore, the challenges of in vivo applications are identified, and strategies to overcome these challenges, such as siRNA delivery methods, biodistribution, and immunotoxicity prevention, are highlighted. Lastly, nucleotide chemical modifications to the ribose sugar and phosphodiester bonds and their effects on siRNA stability, activity, and interaction with the RISC complex are discussed. Overall, this review serves as a guide for well‐designed and rigorously tested siRNA sequences, starting from initial in silico design to the application of siRNA for research or development of siRNA‐based therapeutics.

## 1. Introduction

**Figure 1:**
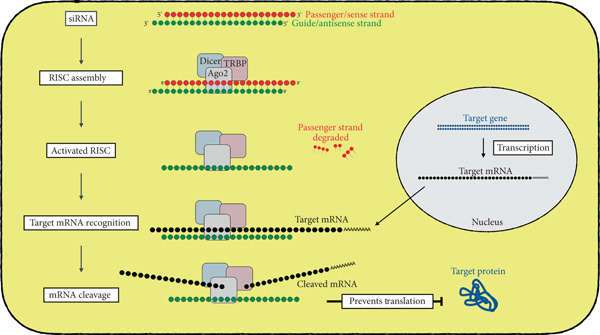
Schematic diagram depicting the mechanism of siRNA‐mediated gene knockdown. The illustration shows the mechanism of siRNA‐mediated knockdown of a target gene (blue) by targeting the mRNA (black). The siRNA with passenger/sense strand (red) and guide/antisense strand (green) is recognized by RNA‐binding proteins Dicer, TRBP, and AGO2. The siRNA and RNA‐binding proteins together form the RNA‐induced silencing complex (RISC). Following the assembly, RISC is “activated” as siRNA unwinds, and the passenger/sense strand is degraded. The guide/antisense strand in the activated RISC recognizes the target mRNA. Ago2 protein in the RISC cleaves the mRNA, thus preventing translation to the target protein.

RNA interference (RNAi) is a posttranscriptional gene regulation mechanism mediated by double‐stranded small interfering RNA (siRNA) or microRNA (miRNA) [1]. In this review, we will focus on siRNA and the mechanism of gene knockdown in mammalian cells (Figure 1). siRNA forms a complex with multiple RNA‐binding proteins, creating an RNA‐induced silencing complex (RISC) [2, 3]. Dicer and TAR RNA–binding protein (TRBP) recognize the thermodynamic asymmetry of siRNA and orient the siRNA into the RISC complex [4–6]. The siRNA strand that has the exact same sequence as the target mRNA is degraded by the RISC and is known as the passenger or sense strand [7]. The other strand, which is retained in the RISC complex, is known as the guide or antisense strand. The guide/antisense binds to the complementary sequence on mRNA, positioning the activated RISC complex at the target mRNA [8–10]. Argonaute2 (AGO2) is the endonuclease of the RISC complex. It cleaves the phosphodiester bond between the 10th and 11th nucleotides of the targeted mRNA sequence, causing the mRNA to be degraded and thus preventing its translation [11, 12].

**Figure 2:**
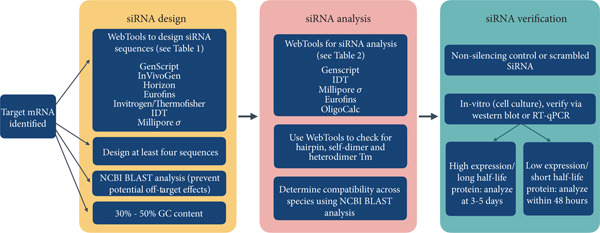
Important aspects of siRNA design, analysis, and verification. Schematic flow chart outlining the workflow for siRNA design, analysis, and verification. The workflow initiates with selecting the target mRNA sequence. siRNA design (yellow panel) involves generating and selecting unique siRNA sequences. The siRNA analysis (red panel) requires analysis of the chosen siRNA sequences for secondary structures, self‐dimerization, and checking compatibility across species for translatability. The siRNA verification (green panel) involves generating a nonsilencing control and verifying siRNA efficacy in vitro before using siRNA for in vivo applications.

RNAi by siRNA and miRNA are similar in that they both recognize specific sequences on target mRNA and prevent its translation. miRNA recognizes and binds to the 3 ^′^ untranslated region (3 ^′^ UTR) of mRNA targets, thereby stalling their translation. miRNA, however, can bind to multiple mRNA targets, limiting its general specificity. In contrast, siRNA targets a single, specific mRNA sequence and cleaves it to promote degradation and prevent translation [13]. Since siRNA is an RNA molecule, it can be easily administered to cells both in vitro and in vivo and can be designed to target virtually any gene. These characteristics make RNAi via siRNA a versatile tool for gene knockdown compared to other gene‐editing tools like miRNA and CRISPR/Cas9. CRISPR/Cas9 also requires integration into the genome and results in permanent changes in the DNA for gene knockout, in contrast to reversible knockdown by siRNA. Due to its versatility, siRNA has been explored as a treatment option for previously drug‐resistant disease conditions [14–16]. Seven siRNA‐based therapeutics are now FDA‐approved for use in clinics, while several others are currently in clinical trials, highlighting the promising future of siRNA‐mediated treatment [17–19]. siRNA is also a powerful research tool for gene knockdown studies. Designing effective siRNA for any target gene is a complex process, starting with in silico analysis and proceeding to in vitro or in vivo applications (Figure 2). This review integrates both theoretical and practical aspects of selecting and testing siRNA, serving as a comprehensive guide for researchers.

## 2. Selecting Target mRNA

The first step in designing an effective siRNA is identifying a suitable target mRNA sequence. The target mRNA sequences can be obtained from the NCBI website (https://www.ncbi.nlm.nih.gov/). The NCBI BLAST tool (http://www.ncbi.nlm.nih.gov/BLAST/) can be used to analyze the splice variants, isoforms, and species specificity of target sequences [20]. Selecting a target sequence conserved across species is most desirable for easy translation among species but is not always possible. Further, if the gene has multiple isoforms, the mRNA variants have to be analyzed to identify a conserved sequence for targeting. In contrast, unique sequences in splice variants can be used to target only specific isoforms [21–23].

The accessibility of the selected target mRNA sequence to the siRNA guide/antisense strand is directly correlated to the efficiency of gene knockdown. Therefore, mRNA regions that are bound to regulatory proteins like the 3 ^′^ and 5 ^′^ untranslated regions (UTRs), the start codon, and the 50–100 bp region downstream of the start codon can be targeted but are usually not recommended [24]. The start codon and the rest of the coding sequence (CDS), which corresponds to the nucleotides that code for amino acids in a protein, are annotated on the NCBI website for all mRNA RefSeqs [25]. The CDS should preferably be used, as opposed to the entire open reading frame (ORF), which also includes the untranslated regions. Lastly, mRNA sequences forming secondary structures can restrict accessibility to the guide/antisense strand on RISC and should be avoided [26, 27].

## 3. siRNA Sequence Design

siRNA sequences must be carefully designed to ensure effective gene knockdown. Online applications (Table 1) generate siRNA sequences for target mRNA using either gene ID, accession number, or FASTA sequence. These applications use different criteria to generate siRNA sequences for a given mRNA sequence. Here, we will discuss the features of siRNA that have been shown to improve its efficacy. These are guidelines for in silico analysis, which can be used to select the best possible siRNA sequences from a list of siRNAs generated by the applications (Table 1). Generally, the siRNA sequences generated are 21 nt (19 + 2) in length, but a few applications allow customization to longer sequences (27–29 nt). In this review, we will discuss the features of 21‐nt siRNA.

**Table 1:** Applications to design siRNA sequences.

**Application**	**Link**
Eurofins	https://eurofinsgenomics.eu/en/ecom/tools/sirna‐design/
GenScript	https://www.genscript.com/design_center.html
Horizon	https://horizondiscovery.com/en/ordering‐and‐calculation‐tools/sidesign‐center
IDT	https://www.idtdna.com/site/order/designtool/index/CRISPRCUSTOM
Invitrogen/ThermoFisher	https://rnaidesigner.thermofisher.com/rnaiexpress/
InVivoGen	https://www.invivogen.com/sirnawizard/
Millipore Sigma	https://www.sigmaaldrich.com/US/en/technical-documents/technical-article/genomics/gene-expression-and-silencing/mission-predesigned-sirna
siDirect	https://sidirect2.rnai.jp

The most effective RNA target sequences have been reported to have an overall GC content between 36% and 52% [28]. siRNA sequence that forms secondary structures such as hairpins or self‐dimers is less likely to be effective at target gene knockdown [29]. The GC content and secondary structure formation can be evaluated based on the selected siRNA sequences using the applications listed in Table 2. siRNA sequences can be entered in these applications, and the programs predict the possibility of potential secondary structures. Ideally, the melting temperature (*T*_*m*_) for internal secondary structure should be less than 20°C; however, *T*_*m*_ values between 20°C and 60°C may be acceptable [28].

**Table 2:** Applications to evaluate siRNA properties (hairpin, self‐dimer, and heterodimer Tm).

**Application**	**Link**
Eurofins	https://eurofinsgenomics.eu/en/ecom/tools/oligo-analysis/
GenScript	https://www.genscript.com/tools/oligo-primer-calculation
IDT	https://www.idtdna.com/pages/tools/oligoanalyzer
MilliporeSigma	https://www.sigmaaldrich.com/US/en/technical-documents/technical-article/genomics/pcr/oligo-evaluator-for-tm-calculation-primer-analysis
Oligocalc	http://biotools.nubic.northwestern.edu/OligoCalc.html

**Figure 3:**
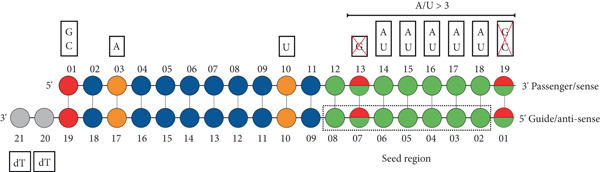
Guidelines for selecting nucleotides at specific positions on siRNA. Schematic diagram depicting desirable nucleotide selection at specific locations on the passenger/sense strand (top strand) and the guide/antisense strand (bottom strand) of siRNA. Each circle represents a nucleotide. The preferred nucleotides for better siRNA efficacy at specific positions are indicated with various colors. Nucleotides G or C (red) are preferred at Position 19, A (orange) at Position 17, and U (orange) at the cleavage site Position 10, and dT overhangs (gray) at the 3 ^′^ end of the guide/antisense strand are preferred for improved siRNA efficacy. The seed region (green) is highlighted with a dotted rectangle. The seed region should contain at least four A/U nucleotides. The remaining nonpreferred nucleotides are represented in blue circles.

The most critical criterion for selecting the best siRNA in silico is an A/U enrichment at Positions 2–8 (5 ^′^ end) of the *guide/antisense strand*; this region is referred to as the seed region. An A/U‐rich seed region significantly enhances the effectiveness of siRNA for on‐target gene knockdown [30, 31]. In addition, a seed region with low *T*_*m*_ (A/U‐rich) minimizes the chances of off‐target effects [32]. Due to the high A/U content, the seed region is thermodynamically less stable (low *T*_*m*_), compared to the rest of the siRNA. This confers a thermodynamic asymmetry in the siRNA, which is another important factor that is predicted to influence the efficacy of siRNA. Asymmetry of siRNA maximizes the likelihood of loading the intended guide/antisense strand into the RISC complex, thus increasing efficacy [6, 33, 34]. Therefore, at least four A or U bases (nonconsecutive) in the seed region are important to provide thermodynamic asymmetry for selection of the intended guide strand and result in an effective on‐target siRNA (Figure 3) [28, 31, 35, 36].

In addition to thermodynamic asymmetry, structural asymmetry is conferred by adding either two deoxythymidine residues (dT–dT) or two ribonucleotide overhangs at the 3 ^′^ end of the guide/antisense strand. Studies have shown that the strand with an overhang is preferentially loaded into the RISC complex [37–39]. Therefore, adding overhangs on the guide/antisense strand increases the likelihood of incorporation on the RISC, leading to an increase in siRNA potency. In addition, nucleotide base preferences at specific locations of the passenger/sense strand that enhance siRNA potency have been identified empirically or via computational modeling. These base preferences are G/C at Position 19, A at Position 17, A/U (but not G/C) at Position 01, and U at Position 10 on the guide/antisense strand (Figure 3), [28, 31, 35]. In addition, siRNA can be labeled with a variety of fluorophores (i.e., fluorescein, Cy3, and Cy5) to detect cellular uptake. The fluorophores should be placed at the 3 ^′^ end of the passenger/sense strand as this usually minimizes the chance of steric hindrance and does not impact the efficacy of gene knockdown [40, 41]. Designing siRNA that fulfills all the abovementioned criteria can be challenging and is not always possible. Therefore, multiple siRNA sequences that match most of the criteria should be selected for further evaluation in vitro.

## 4. Validation of siRNA In Vitro

siRNA sequences should be evaluated in vitro before being used for in vivo applications, if possible. To achieve effective gene silencing, it is recommended that four to five siRNA sequences complementary to different regions of the target mRNA be tested. Sometimes, a combination of two or more siRNAs can be more effective than a single siRNA [42]. siRNA efficacy can be assessed primarily by measuring the level of the target protein or mRNA knockdown using Western blot or RT‐qPCR, respectively. A knockdown of 70% or higher at the protein level is considered standard for effective siRNA gene silencing [35, 43]. However, for certain types of target genes, a moderate knockdown (40%–50%) may be more relevant. For example, proteins that have a long half‐life are more difficult to knock down and may take substantially longer using siRNA [44, 45]. Another example is targeting mRNAs that code for modulatory proteins, such as transcription factors like HIF‐1*α* and BCL‐1. Modulatory proteins influence the expression of several other target genes, and small alterations in gene expression of these modulatory proteins can impact downstream function. Therefore, silencing of modulatory proteins by 70% or more could disrupt the homeostasis in the cell, and a lower level of knockdown may be warranted and more relevant.

If the desired knockdown is achieved, secondary assessments should be conducted to validate gene silencing. These secondary assessments include analyzing the target protein′s function/activity (e.g., enzyme activity assay) or evaluating downstream protein levels [46]. Another method for validating siRNA knockdown is performing rescue experiments. Rescue experiments are conducted by overexpressing the target gene that has either silent point mutations (at least three) in the siRNA binding region or another mismatch that does not affect protein function but prevents siRNA binding [47]. In cell culture, a range of siRNA concentrations from 5 to 100 nM should be tested to confirm the specificity of the selected siRNA [48, 49]. A 70% knockdown with 20 nM or less siRNA is considered optimal [35, 43]. These rigorous secondary assessments are crucial, especially for siRNA that targets regulatory genes or those that exhibit moderate levels of knockdown, to validate targeted gene silencing and eliminate the possibility of off‐target effects [50].

Nonsilencing siRNA controls (NSCs) should be included to assess potential off‐target effects. Ideally, NSC sequences are generated by modifying sequences of the selected siRNA by changing just three or four individual nucleotides or by completely scrambling the sequence [42, 51]. In either case, the resulting NSC sequence should be analyzed via the NCBI BLAST tool (http://www.ncbi.nlm.nih.gov/BLAST/) to ensure that it is not complementary to any endogenous mRNA expressed in the cell. siRNA sequences that target mRNA that is not present in the mammalian transcriptome, such as green fluorescent protein (GFP), could also be used [52].

## 5. siRNA Silencing in Three‐Dimensional (3D) Cell Culture

Techniques for culturing cells in 3D use biomaterials such as hydrogel and bioscaffolds, in contrast to traditional monolayer 2D cultures [53]. 3D cell cultures better simulate the tissue microenvironment compared to 2D cultures, as they effectively mimic the structural and biochemical complexities of native tissues, including interactions with the extracellular matrix [54]. Studies have shown that 3D cell cultures are more resistant to drug responses compared to 2D cell cultures [55]. Drug resistance arises from factors like concentration gradient issues through the extracellular matrix. In organoid culture, peripheral cells likely encounter higher drug levels than cells at the center, compared to a more uniform distribution in 2D monolayer culture [56]. This principle is especially important when the delivery mechanism is dependent on endocytosis, such as in the case of siRNA delivery through lipid nanoparticles. *Another challenge is the requirement for higher drug doses in 3D cultures. A previous study showed that a 3D culture of human lung carcinoma A549 cells required 6600 times more chemotherapeutic drug treatment compared to a monolayer culture to achieve the same effect* [57]. siRNA is no exception; siRNA delivery using traditional lipid–siRNA complex–based transfection is not efficient in delivering siRNA to a 3D cell culture [58, 59]. To address this issue, a study utilized serum‐containing media for preparing the siRNA–lipid complex instead of the conventional low‐serum media (Opti‐MEM) used in 2D cultures. This approach improved siRNA delivery, resulting in transient gene suppression in a 3D cell culture model [59]. Studies have attempted to utilize delivery methods akin to in vivo siRNA applications to enhance siRNA efficacy in 3D cell culture models. For example, using a lipid‐like molecule or lipidoid, 98N_12_, to prepare the siRNA complex resulted in up to 90% knockdown in 3D scaffolds [60]. Noske et al. [61] compared multiple lipids and polyethyleneimine (PEI)‐based linear or branched nanoparticles for siRNA delivery in a 3D air–liquid interface culture. Modified PEIs showed reduced delivery and less siRNA‐mediated knockdown in 3D versus 2D cell culture in the same cell line [58, 61]. Overall, siRNA delivery and subsequent gene knockdown in 3D cell cultures and advanced 3D models like organoids do not align with the mechanisms of 2D cell cultures. The delivery of siRNA in organoid cultures and effective gene knockdown pose further challenges. Efficient use of siRNA and other genome‐modifying tools in 3D organoids is discussed elsewhere [62]. More research is required to develop an efficient siRNA delivery mechanism for 3D cell culture systems.

## 6. siRNA Applications In Vivo

All of the verification methods described for in vitro applications are also helpful for in vivo applications. However, additional challenges in vivo include an assessment of the immune response, siRNA stability in serum, and biodistribution. Strategies to overcome these challenges include chemical modifications, targeted delivery conjugates, and delivery via nanoparticles. Chemical modifications of siRNA can help overcome these challenges by either enhancing metabolic stability or evading immunotoxicity, leading to increased efficacy and durability of siRNA‐mediated knockdown in vivo (see the Types of siRNA Chemical Modifications section).

The stability of siRNA in vivo can be compromised by endo‐ and exonucleases and phosphatases, which can rapidly degrade siRNA activity when using unmodified siRNA. A study demonstrated that unmodified and unencapsulated siRNA starts degrading within 3–6 h after in vivo administration [63]. Further, encapsulation of siRNA into a delivery mechanism like lipid nanoparticles or peptide conjugates further protects siRNA from degradation via nucleases in the serum [63, 64].

The mammalian immune system is programmed to recognize dsRNA and trigger an immune response as a mechanism to combat a dsRNA virus infection. Long double‐stranded RNAs (> 30 bp) are detected by dsRNA‐dependent protein kinase (PKR), toll‐like receptors (TLRs), and retinoic acid–inducible gene 1 (RIG‐I) receptors, which eventually lead to an immune response by innate immune cells [65–67]. The use of short 21‐nt siRNA avoids activation of an immune response [68, 69]. However, specific RNA sequence motifs such as 5 ^′^‐GUCCUUCAA‐3 ^′^ or 5 ^′^‐UGUGU‐3 ^′^ can activate the innate immune system and therefore should be avoided to prevent immunotoxicity [70–72].

The biodistribution of siRNA in animal models is dependent on the route of administration and mode of siRNA delivery [73]. When administered systemically via subcutaneous, intraperitoneal, or intravenous injection, nontargeted siRNA predominantly accumulates in organs such as the liver, kidney, heart, and reticuloendothelial systems (RESs) [73–75]. In addition, targeted siRNA delivery can also be achieved by conjugating siRNA to molecules/peptides that bind to receptors present only on target organs. Examples of this are the *N*‐acetylgalactosamine (GalNAc)‐conjugated siRNAs that have shown the most promise in treating liver‐associated diseases [76–78] and peptide‐conjugated nanoparticles that specifically target the placenta [79, 80]. Targeted delivery of siRNA to specific organs or tissues significantly improves siRNA efficacy by abating the potential off‐target effects [73, 81–83]. Delivery mechanisms using nanoparticles significantly improve the stability and cellular uptake of siRNA in vivo [84]. Lipid‐ and polymer‐based nanoparticles are the preferred delivery systems for siRNA as they provide protection from degradation and are time‐released [85, 86]. The surface of these nanoparticles can also be conjugated with antibodies or peptides for tissue or cell‐specific targeted delivery [87]. Delivery mechanisms using viral vectors such as lentivirus, adenovirus, and adeno‐associated virus require conversion of the siRNA sequence to a short hairpin RNA (shRNA) [88, 89]. The siRNA sequences are converted to shRNAs by introducing a hairpin loop [23, 90]. The nucleotide number and sequence of the shRNA hairpin loop can have a significant impact on gene knockdown. Therefore, the shRNA loop needs to be carefully designed, and the specifics of this process are discussed elsewhere [91–93].

The in vivo applications of siRNA are extensive, encompassing gene knockdown for basic research, analysis of signaling pathways, and potential therapeutic uses. siRNA can also target non‐mRNA nuclear RNA to study their physiological function. For instance, a study targeted the U1 small nuclear RNA (snRNA) by siRNA in macrophages and identified a physiological role of U1 snRNA in innate immune system regulation [94]. Another use of siRNA involves targeting viral RNA, impairing virus replication, and effectively eradicating the infection. Clinical trials are ongoing for siRNA targeting SARS‐CoV‐2 and respiratory syncytial virus, with additional siRNA therapies in development [95–98]. Lastly, five siRNA therapies have received approval from the FDA and are currently used for the treatment of diseases that were previously resistant to medication [99]. Four of these five siRNAs are conjugated with tertiary GalNAc to target the liver. Research is underway to develop siRNA therapies for various organs, including the eyes, kidneys, brain, and skin [73]. In conclusion, siRNA has emerged as a powerful tool in gene silencing with promising applications in fundamental as well as clinical research.

## 7. Types of siRNA Chemical Modifications

Nucleotide chemical modifications for siRNA are categorized into (1) ribose sugar modifications, (2) phosphate‐linker modifications, and (3) nucleotide base modifications. Chemical modifications of siRNA offer more flexibility in selecting the mRNA target sequence, as they no longer need the conventional sequence‐dependent thermodynamic asymmetry. Structural asymmetry can also be achieved through chemically modified substitutes, providing higher or lower steric hindrance. Therefore, the parameters for determining the chemically modified siRNA sequence vary significantly from those of unmodified siRNA [100].

Modifications to the ribose sugar, such as replacing the 2 ^′^‐OH group with a 2 ^′^‐OMe (methyl), 2 ^′^‐F (fluoro), or 2 ^′^‐MOE (methoxyethyl) group, are effective at inhibiting endonuclease activity and increasing siRNA stability in serum [101, 102]. The 2 ^′^‐F group most closely resembles the 2 ^′^‐OH group and is therefore well tolerated in the guide/antisense strand, especially at Positions 9–11 counting from the 5 ^′^ end [103–105]. In contrast, the bulkier 2 ^′^‐OMe‐ and 2 ^′^‐MOE‐modified substitutes cause steric hindrance when placed at certain positions on the guide/antisense strand. Therefore, modification with 2 ^′^‐OMe and 2 ^′^‐MOE at nucleotides at Positions 2 and 14 on the guide/antisense strand is often avoided to limit steric hindrance [100, 103, 106]. Modifications of the ribose cyclic structure, such as locked nucleic acid (LNA), unlocked nucleic acid (UNA), and glycol nucleic acid (GNA), have also been shown to have the potential to enhance siRNA performance [107–109].

**Figure 4:**
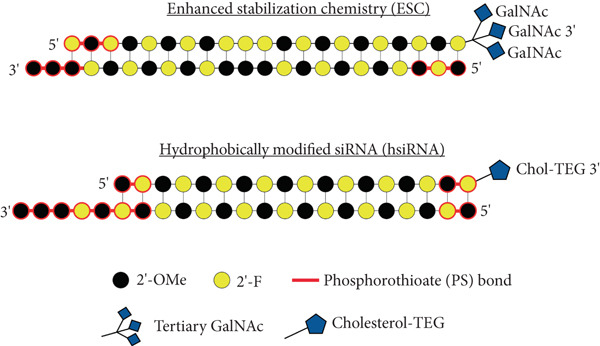
Schematic diagram of fully chemically modified siRNAs. Two siRNA chemical modification strategies, enhanced stabilization chemistry (ESC) siRNA and hydrophobically modified siRNA (hsiRNA), are illustrated. For both siRNAs, the top strand represents the passenger/sense strand, and the bottom strand represents the guide/antisense strand. Nucleotide chemical modifications include 2 ^′^‐OMe‐modified nucleotides (black), 2 ^′^‐F‐modified nucleotides (yellow), and phosphorothioate bond (red). The ESC siRNA is conjugated with tertiary *N*‐acetylgalactosamine (GalNAc) for liver‐specific delivery, and the hsiRNA is conjugated with cholesterol+TEG linker (Chol‐TEG) for easy uptake in other organs in vivo.

The replacement of phosphodiester bonds with phosphorothioate (PS) bonds prevents exonuclease‐mediated degradation of siRNA. Replacing the phosphodiester bonds with PS linkages at all siRNA nucleotides negatively impacts the siRNA activity and induces off‐target effects [110, 111]. The PS linkages are often limited to terminal nucleotides on both strands of siRNA (Figure 4). Additionally, PS linkages are chiral, and siRNA synthesized with PS linkages will have a mixture of *Rp* and *Sp* diastereomers, potentially affecting their interaction with the RISC complex [112, 113]. Therefore, phosphorodithioate (PS2) linkages at terminal nucleotides are preferred to reduce stereochemical complexity and enhance gene silencing activity compared to siRNA with PS linkages [114].

A 5 ^′^ phosphate group on the guide/antisense strand is important for loading onto the RISC complex [11, 115, 116]. Adding a 5 ^′^ phosphate group improves the potency of siRNA in vitro [117]. However, siRNA with a 5 ^′^ phosphate group in animal serum is dephosphorylated within 2 h. 5 ^′^‐E‐vinyl phosphonates (5 ^′^‐VPs) are a substitute for the 5 ^′^ phosphate group that improves siRNA stability by avoiding the dephosphorylation in serum and increasing RISC loading. Therefore, substitution of 5 ^′^ phosphate with 5 ^′^‐VP on siRNA is recommended for in vivo applications [117–119].

For in vivo applications, fully modified siRNA, with all nucleotides chemically modified by substituting 2 ^′^‐OH with either 2 ^′^‐OMe or 2 ^′^‐F, has enhanced RISC loading, provides better tissue distribution, and results in productive mRNA silencing compared to partially modified or naked siRNA [120]. Several pharmaceutical companies have described fully chemically modified siRNA templates. The siRNA modification patterns established by these companies are Standard Template Chemistry, Enhanced Stabilization Chemistry (ESC), Advanced ESC, and ESC Plus (ESC+). These siRNA chemistries vary in antisense length, PS‐linkage location, and the 2 ^′^‐F/2 ^′^‐OMe patterns, detailed in [121].

The ESC siRNA, conjugated with GalNAc, is commonly utilized for both in vitro and in vivo studies targeting the liver or hepatic cell lines [112, 122, 123]. The ESC siRNA is 23 nt and contains 2‐nt overhangs at the 3 ^′^ end of the guide/antisense strand. In addition, the phosphodiester bond between the three terminal nucleotides at the 5 ^′^ end of the guide/antisense strand and the 3 ^′^ end of the guide/antisense and passenger/sense strand is replaced by PS linkages (Figure 4). Another commonly used fully modified siRNA, also known as self‐delivering siRNA (sd‐rxRNA) or hydrophobically modified siRNA (hsiRNA), is a cholesterol‐conjugated asymmetrical siRNA with 5‐nt overhangs at the 3 ^′^ end of the guide/antisense strand and has comparably more PS linkages compared to ESC siRNA (Figure 4). This type of siRNA is readily taken up by cells without a transfecting agent in vitro. It also accumulates in tissues not targeted by liver‐specific GalNAc‐conjugated siRNA, such as the kidney, spleen, lung, heart, muscle, and placenta [111, 124]. If administered locally, the hsiRNA can also be taken up in the central nervous system and eyes [125, 126]. The chemistry of the possible modifications and their effect on siRNA efficacy has been reviewed extensively and will not be covered here [127–129].

## 8. Conclusion

siRNA design is critical for achieving optimal gene knockdown. Designing an effective siRNA begins with selecting a suitable target mRNA sequence. The siRNA sequences can be designed using online applications. The siRNA sequences with appropriate GC content, asymmetry, and minimal secondary structure formation should be selected for best performance. Most importantly, the seed region of the siRNA should be A/U‐rich to achieve optimal gene knockdown. The efficacy of the selected siRNA should be assessed by measuring protein or mRNA knockdown in vitro. Secondary assessments, such as dose response and rescue experiments, should be performed to validate siRNA‐mediated gene knockdown. In vivo siRNA applications are faced with additional challenges, such as the potential to induce an immune response, siRNA stability, and biodistribution. These challenges can be overcome by using siRNA encapsulated in nanoparticles, targeting siRNA delivery, and/or using chemically modified siRNA. Overall, rigorous siRNA design is essential for successful gene knockdown and optimal efficacy.

siRNA is a valuable research tool with numerous practical applications across various fields. It is employed in fundamental research to investigate gene function through the selective silencing of specific genes. Strategies determining the ideal nucleotide sequence for siRNA discussed in this review pertain to the mammalian RISC complex. However, the RISC proteins and RNAi mechanisms exhibit differences across different species [130, 131]. Consequently, employing siRNA in alternative model organisms such as yeast, zebrafish*, Drosophila melanogaster*, plants, or microbes may necessitate further evaluation. In summary, siRNA shows significant potential as both a research and therapeutic tool. However, overcoming these challenges and investigating its use in various biological systems are crucial for exploring its full capabilities.

siRNA technology has considerably advanced in recent years, especially as a therapeutic. Chemical modifications of siRNA have increased its potential to overcome challenges such as instability, immune reactions, and off‐target effects, which previously hindered its in vivo and clinical applications. Additionally, the specificity of siRNA for a particular gene enhances its suitability for therapeutic purposes. siRNA demonstrates clinical applications in combating diseases that can be prevented or eradicated by silencing gene expression [132, 133]. Given the increasing demand for personalized and precision medicine, siRNA‐based therapies are becoming competitive with other gene‐altering technologies for diseases caused by genetics. A few instances where the siRNA therapy is beneficial over the irreversible genome editing include targeting specific mRNA splice variants, infectious RNA (targeting bacterial or viral transcripts), and RNA molecules other than mRNA [75]. Current advancements are focused on making siRNA more accessible to target organs other than the liver by means of conjugations, localized delivery, and more. The rising approval rate of siRNA drugs by the FDA in recent years indicates the growing potential of siRNA therapeutics (Table 3), which are expected to become more mainstream treatments for genetically caused diseases.

**Table 3:** FDA‐approved siRNA drugs. IV, intravenous; SC, subcutaneous.

**Brand**	**siRNA therapeutic**	**Disease target**	**Route**	**Dose**	**Delivery system**	**Citation**
Onpattro	Patisiran	Hereditary transthyretin‐mediated amyloidosis	IV	0.3 mg/kg every 3 weeks	LNP	[134, 135]
Givlaari	Givosiran	Acute hepatic porphyria	SC	2.5 mg/kg every month	GalNAc	[136, 137]
Oxlumo	Lumasiran	Primary Hyperoxaluria Type 1	SC	3 mg/kg every month if under 10 kg, every 3 months if over 10 kg	GalNAc	[138, 139]
Leqvio	Inclisiran	Primary hypercholesterolemia	SC	284 mg every 3 months for the first two doses and then every 6 months	GalNAc	[140, 141]
Amvuttra	Vutrisiran	Hereditary transthyretin‐mediated amyloidosis	SC	25 mg every 3 months	GalNAc	[142, 143]
Rivfloza	Nedosiran	Primary Hyperoxaluria Type 1	SC	128 mg every month if < 50 kg and 160 mg every month if > 50 kg	GalXC RNAi	[144, 145]
Qfitlia	Fitusiran	Hemophilia A or B	SC	50 mg every 2 months, adjust dose and/or interval to maintain antithrombin activity between 15% and 35%	GalNAc	[146, 147]

## Conflicts of Interest

The authors declare no conflicts of interest.

## Funding

No funding was received for this manuscript.

## Data Availability

Data availability is not applicable as this is a review article and no new data were created or analyzed.
